# Association of Transition Zone Burden With Recurrence in Elderly Patients With Paroxysmal Atrial Fibrillation Without Low‐Voltage Areas

**DOI:** 10.1002/clc.70435

**Published:** 2026-08-31

**Authors:** Yuxuan Wang, Yue Qiu, Haojun Wang, Chang Cui, Zidun Wang, Xiaohong Jiang, Mingfang Li, Juejin Wang, Hongwu Chen, Minglong Chen

**Affiliations:** ^1^ Division of Cardiology The First Affiliated Hospital with Nanjing Medical University Nanjing Jiangsu China; ^2^ Division of Cardiology The Affiliated Taizhou People's Hospital of Nanjing Medical University Taizhou Jiangsu China; ^3^ Key Laboratory of Targeted Intervention of Cardiovascular Disease, Collaborative Innovation Center for Cardiovascular Disease Translational Medicine, and Department of Physiology Nanjing Medical University Nanjing China

**Keywords:** atrial fibrillation, left atrial conduction velocity, low‐voltage area, pulmonary vein isolation, transition zone

## Abstract

**Background:**

Transition zone (TZ) may serve as a potential substrate for atrial fibrillation (AF). This study aims to investigate the relationship of TZ burden with left atrial conduction velocity (LACV) and recurrence after circumferential pulmonary vein isolation (CPVI) in elderly patients with paroxysmal AF without low‐voltage areas (LVA).

**Methods:**

Patients with paroxysmal AF without LVA were categorized into three groups based on the TZ burden (< 5%, 5%–15% and > 15%). TZs were defined as regions with bipolar voltage between 0.5 and 1 mV in more than 3 adjacent points. TZ burden was calculated as the proportion of TZ on the entire left atrium surface. LACV were calculated as conduction distance divided by conduction time. Recurrence was defined as any episode of atrial tachyarrhythmia (ATA) lasting ≥ 30 s after a 3‐month blanking period.

**Results:**

Among the 158 enrolled patients (mean age 69.7 ± 3.8 years), 48 (30.4%) had a TZ < 5%, 66 (41.8%) had a TZ of 5%–15%, and 44 (27.8%) had a TZ > 15%. Patients with higher TZ burden exhibited significantly decreased anterior LACV (1.52 ± 0.36 vs. 1.21 ± 0.27 vs. 1.02 ± 0.25 m/s, *p* < 0.0001). After a mean follow‐up of 24.9 months, 40 (25.3%) patients experienced recurrence. Patients with recurrence had higher TZ burden (15.4 [7.2–22.4] vs. 8.2 [3.5–14.3], *p* < 0.0001). TZ burden remained significantly associated with recurrence in a multivariable Cox regression model (adjusted HR 1.065 [95% CI 1.034–1.098], *p* < 0.0001).

**Conclusions:**

TZ burden is correlated with slow LACV and independently predicts ATA recurrence following CPVI.

## Graphical abstract

Transition zone (TZ) is defined as regions with bipolar voltage between 0.5 and 1 mV in left atrium. In 158 elderly PAF patients without low‐voltage areas (LVA), TZ burden is associated with reduced left atrial conduction velocity (LACV) and independently predicts atrial tachyarrhythmia (ATA) recurrence following CPVI.

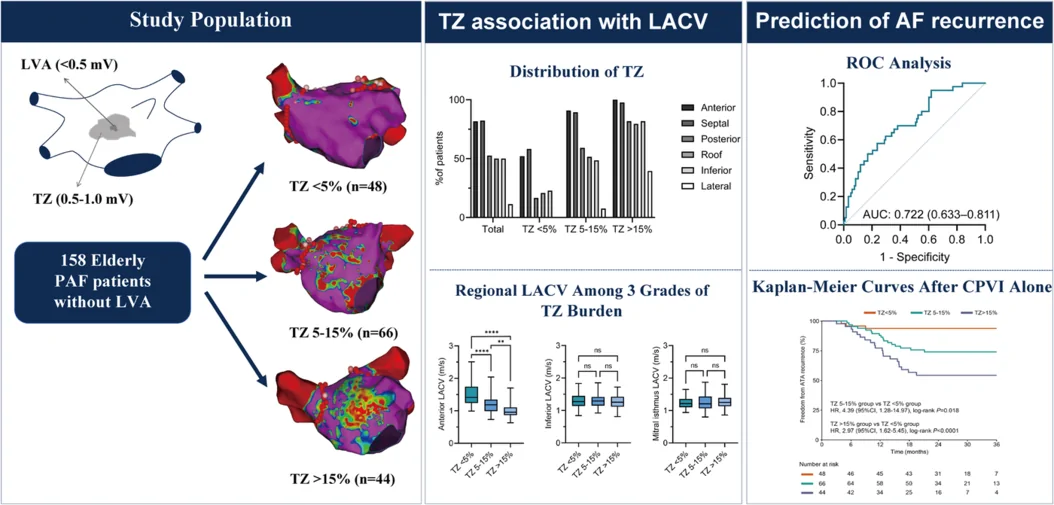

**Abbreviations**
AF: atrial fibrillation
ATA: atrial tachyarrhythmia
CPVI: circumferential pulmonary vein isolation
LA: left atrium
LACV: left atrial conduction velocity
LAT: local activation time
LVA: low‐voltage area
TZ: transition zone

## 1 Introduction

Atrial fibrillation (AF) is the most common arrhythmia [1]. Catheter ablation has become a first‐line rhythm control strategy for AF, with circumferential pulmonary vein isolation (CPVI) serving as its cornerstone [2, 3]. Atrial fibrosis, characterized by local electrical decoupling and heterogeneous slow conduction [4, 5], plays a crucial role in the occurrence and maintenance of AF [6, 7, 8]. It can be identified as low‐voltage area (LVA) on a 3‐dimensional voltage map [9, 10]. LVA, typically defined as region with local bipolar voltage < 0.5 mV [11, 12, 13], has been widely considered as a powerful predictor of AF recurrence following CPVI [13, 14, 15]. Our prior study demonstrated that CPVI plus additional LVA modification significantly reduced long‐term recurrence rates in elderly patients with paroxysmal AF compared to CPVI alone [16]. However, clinical experience indicates that a subset of patients without LVA are still prone to recurrence after CPVI alone. Notably, both electroanatomic and histological evidence suggest that atrial fibrosis is a diffuse rather than focal process [6, 17, 18]. Hence, a single voltage threshold is insufficient to distinguish between diseased and healthy regions.

Transition zone (TZ) is described as an intermediate state between normal and diseased tissue [10, 19, 20, 21]. Although the voltage cutoffs defining TZ vary modestly using different mapping systems [10, 20], TZ was highly associated with abnormal electrograms, suggesting it as a potential arrhythmogenic substrate [10, 20]. However, the predictive value of TZ for post‐CPVI recurrence remains unvalidated. Only a few studies explored the relationship between the presence of TZ and recurrence, showing inconsistent results [20, 21]. We hypothesized that TZ is a predictor of AF recurrence, and its impact on the outcomes following CPVI is quantitative rather than qualitative. The aims of this study are: (1) to investigate the relationship of TZ burden and recurrence following CPVI in elderly patients with paroxysmal AF without LVA; and (2) to evaluate the association of TZ burden with left atrial conduction velocity (LACV).

## 2 Methods

### 2.1 Patient Population

**Figure 1:**
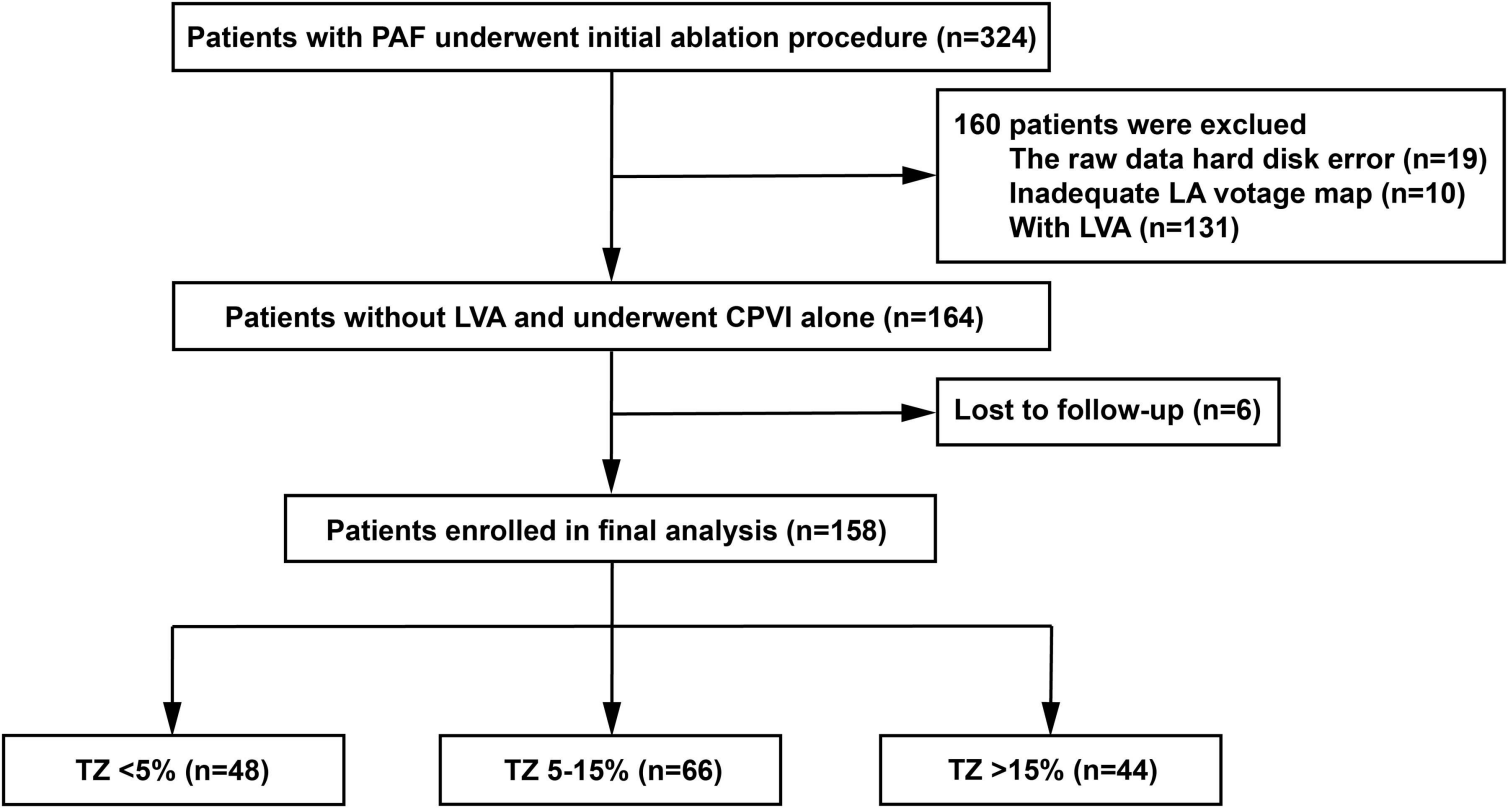
Study flow‐chart. CPVI, circumferential pulmonary vein isolation; LA, left atrium; LVA, low‐voltage area; PAF, paroxysmal atrial fibrillation; TZ, transition zone.

**Figure 2:**
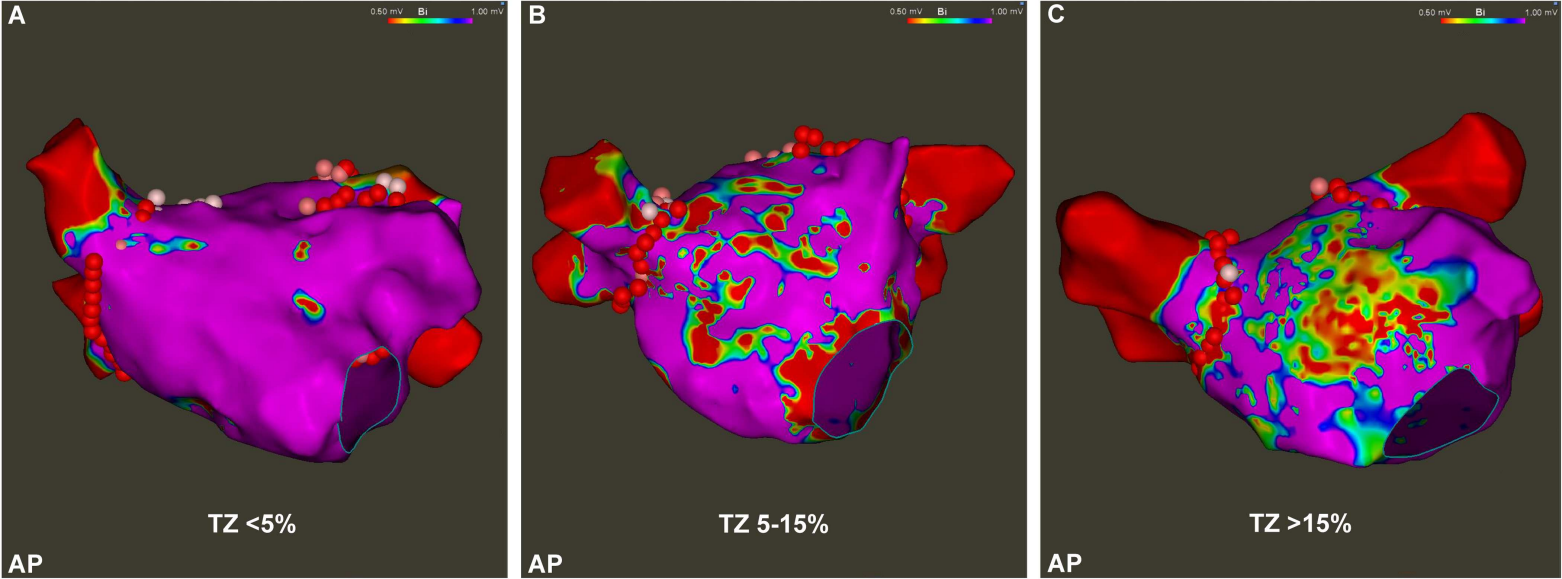
The LA bipolar electroanatomic voltage map of patients without LVA (< 0.5 mV) with varying degree of TZ (0.5–1 mV) burden undergoing CPVI only. (A) A representative patient with slight TZ burden (< 5%). (B) A representative patient with moderate TZ burden (5%–15%). (C) A representative patient with severe TZ burden (> 15%). CPVI, circumferential pulmonary vein isolation; LA, left atrium; LVA, low‐voltage area; TZ, transition zone.

Patients aged 65−80 years with paroxysmal AF undergoing their first‐time catheter ablation at our center between January 2019 and December 2021 were enrolled. The study flow‐chart is shown in Figure 1. The exclusion criteria were prior catheter ablation or cardiac surgery, absence of data, incomplete voltage map, with LVA, additional ablation, and lost to follow‐up. The enrolled patients were categorized into three groups based on the TZ burden (< 5%, 5%–15%, and >15%, Figure 2). The study was approved by the Ethics Committee of The First Affiliated Hospital of Nanjing Medical University, and all patients provided written informed consent.

### 2.2 Ablation Procedure

All antiarrhythmic medications were discontinued for a minimum of five half‐lives and amiodarone for 2 months before the procedure. Electrophysiological studies were conducted following overnight fasting and mild sedation induced by intravenous midazolam and fentanyl. Mapping and ablation were guided by a 3D mapping system (Biosense Webster), utilizing a multipolar mapping catheter and an irrigated radiofrequency catheter with smart‐touch contact force capabilities. CPVI was performed guided by ablation index values (power 30–40 W, contact force 5–30 g), with targets of 500 for the anterior, 400–450 for the roof, and 350–400 for the inferior and posterior segments. Concomitant atrial flutter or other tachycardias were ablated during the procedure, and sinus rhythm was restored by electrical cardioversion if AF persisted after CPVI.

### 2.3 Voltage Map

After CPVI, a detailed 3‐D voltage map of the left atrium (LA) (recorded surface points of at least 350) was created using a multipolar mapping catheter (Pentrary, Biosense Webster Inc., Diamond Bar, CA, USA) and CARTO3 System (Biosense Webster Inc., Diamond Bar, CA, USA) during sinus rhythm. LVA were defined as regions with bipolar electrogram amplitude < 0.5 mV, with TZs as 0.5–1 mV in more than 3 adjacent low‐voltage points with a space difference of 0.5 cm [21]. LA was divided into six segments: Anterior, septal, posterior, roof, inferior, and lateral wall [22]. LVA burden and TZ burden were calculated as their respective percentage of the entire LA surface area (excluding the PV ostia and mitral valve area).

### 2.4 LACV Measurement

Isochronal activation maps of LA were created with 5‐ms intervals to calculate local activation time (LAT) and LACV. LAT was manually annotated based on the maximum slope (dV/dT) of bipolar and unipolar signals during sinus rhythm, ensuring consistency with surrounding electrograms. LACV was determined in the direction of wavefront propagation (least isochronal crowding) and calculated as the distance along the 3‐D model surface between two points divided by the LAT difference, expressed in m/s. Mean LACV for each region was determined by averaging the conduction velocity between 3 pairs of points [23, 24, 25].

**Figure 3:**
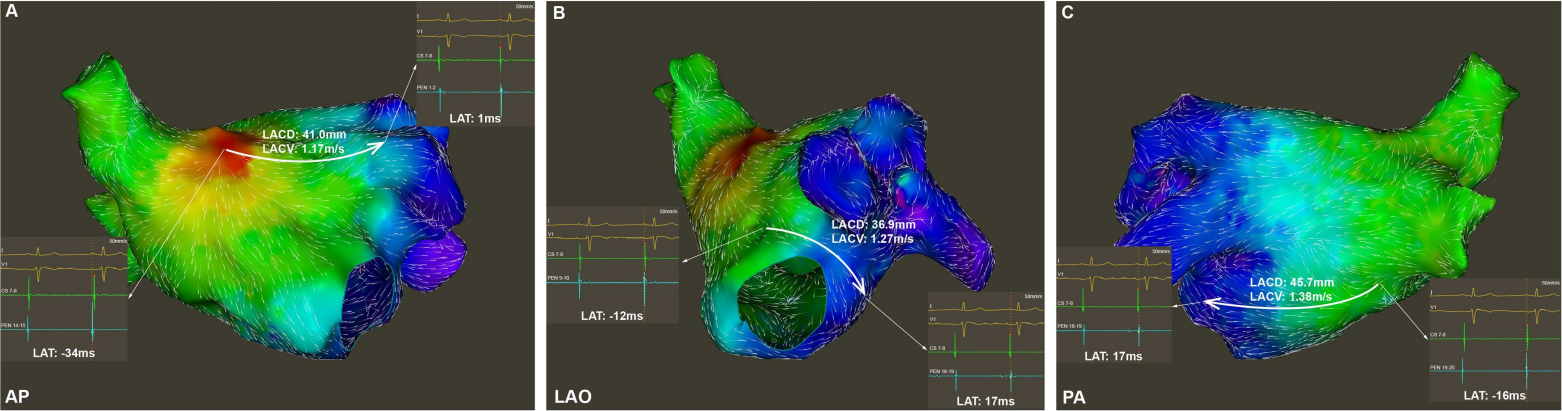
LACV measurement. The conduction time was calculated as the difference in LAT. LACV was determined in the direction of wavefront propagation and calculated as the distance between the start and end points divided by the conduction time. (A) Anterior LACV measurement. (B) Mitral isthmus LACV measurement. (C) Inferior LACV measurement. AP, anteroposterior; LACD, left atrial conduction distance; LACV, left atrial conduction velocity; LAO, left anterior oblique; LAT, local activation time; PA, posteroanterior.

We measured the conduction velocity of left atrial activation wavefront along three distinct pathways: anterior, mitral isthmus, and inferior. The anterior pathway originated at the Bachmann bundle insertion, traversed the anterior wall, and ended at the left atrial appendage orifice. The mitral isthmus pathway started at the earliest activation site within the mitral isthmus, propagated along the isthmus, and terminated at its latest activation point. The inferior wall pathway began at the earliest activation point on the posterior septal surface, crossed the inferior wall, and ended at the latest activation site in the mitral isthmus region, as illustration in Figure 3.

### 2.5 Follow Up

All patients received anti‐arrhythmic drugs during the first 3 months post‐ablation unless contraindicated. Anticoagulation was administered for a minimum of 3 months following the procedure and at the discretion of the operators thereafter. Follow‐up assessments included surface ECG and 24‐h Holter monitoring at 1, 3, and 6 months, and a 7‐day Holter recording or 24‐h Holter monitoring at 12 months. In subsequent years, clinic visits and 24‐h Holter monitoring were conducted every 6 months, with ECGs performed as needed for symptomatic arrhythmias. Recurrence was defined as any episode of atrial tachyarrhythmia (ATA) lasting ≥ 30 s after the 3‐month post‐ablation blanking period.

### 2.6 Statistical Analysis

Continuous variables were expressed as mean ± standard deviation (SD) or median (interquartile range) and compared using the Student's *t*‐test or Mann−Whitney *U* test. ANOVA or Kruskal−Wallis test was employed for comparisons of continuous variables across three groups, as appropriate. Categorical variables were presented as numbers and percentages and analyzed using Pearson's chi‐square test. Partial correlation analysis was performed to assess the relationship between regional TZ burden and LACV. Univariate and multivariable COX analyses were performed to investigate the relationship between clinical variables and recurrence. Cox regression models were employed to evaluate the effect of TZ burden on ATA recurrence, with the models constructed using incremental adjustments. Freedom from ATA recurrence among the TZ < 5%, 5%–15%, and >15% groups was evaluated using Kaplan‐Meier survival analysis and the log‐rank test. A receiver‐operating characteristic (ROC) curve was used to analyze the sensitivity and specificity of TZ burden in predicting recurrence after CPVI. A two‐sided *p* < 0.05 was considered statistically significant for all analyses. Statistical computations were performed using R software (version 3.3.0).

## 3 Results

### 3.1 Baseline Characteristics

A total of 158 patients (mean age 69.7 ± 3.8 years, 61.4% male) with paroxysmal AF were enrolled in the study, 48 (30.4%) of which had a TZ < 5%, 66 (41.8%) had a TZ of 5%–15%, and 44 (27.8%) had a TZ > 15%. Patients with a higher TZ burden grade were significantly more likely to be female and have larger left atrial diameter (LAD). Patients with TZ of 5%–15% had a higher left ventricular ejection fraction (LVEF) than the other two groups. There were no differences in other baseline characteristics among the 3 groups (Table 1).

**Table 1:** Baseline characteristics of the study population.

Characteristics	Total (*n* = 158)	TZ < 5% (*n* = 48)	TZ 5%–15% (*n* = 66)	TZ > 15% (*n* = 44)	*p* value
Female, *n* (%)	61 (38.6)	12 (25.0)	26 (39.4)	23 (52.3)	0.027
Age, years	69.7 ± 3.8	69.4 ± 3.6	69.8 ± 4.1	70.0 ± 3.8	0.723
BMI, kg/m^2^	24.6 ± 3.3	25.0 ± 4.1	24.5 ± 2.4	24.3 ± 3.3	0.503
AF history, months	12.0 (5.0, 39.0)	12.0 (3.0, 45.0)	12.0 (6.0, 36.0)	24.0 (3.0, 82.5)	0.711
*Comorbidities, n (%)*					
Hypertension	107 (67.7)	30 (62.5)	45 (68.2)	32 (72.7)	0.574
Diabetes	22 (13.9)	5 (10.4)	11 (16.7)	6 (13.6)	0.634
CAD	29 (18.4)	7 (14.6)	10 (15.2)	12 (27.3)	0.198
Stroke or TIA	15 (9.5)	7 (14.6)	3 (4.5)	5 (11.4)	0.172
Congestive heart failure	1 (0.6)	0 (0)	0 (0)	1 (2.3)	0.280
COPD	0 (0)	0 (0)	0 (0)	0 (0)	
OSAS	2 (1.3)	1 (2.1)	1 (1.5)	0 (0)	1
*CHA_2_DS_2_‐VASc score*					0.090
1, *n* (%)	35 (22.2)	17 (35.4)	13 (19.7)	5 (11.4)	
2, *n* (%)	63 (39.9)	14 (29.2)	26 (39.4)	23 (52.3)	
3, *n* (%)	39 (24.7)	12 (25.0)	20 (30.3)	7 (15.9)	
>3, *n* (%)	21 (13.3)	5 (10.4)	7 (10.6)	9 (20.5)	
LAD, mm	37.7 ± 5.2	36.0 ± 5.3	38.0 ± 4.5	39.0 ± 5.6	0.016
LVEF, %	61.8 ± 5.2	61.1 ± 5.0	63.0 ± 5.2	60.8 ± 5.3	0.043

Abbreviations: AF, atrial fibrillation; BMI, body mass index; CAD, coronary artery disease; COPD, chronic obstructive pulmonary disease; LAD, left atrial diameter; LVEF, left ventricular ejection fraction; OSAS, obstructive sleep apnea syndrome; TIA, transient ischemic attack; TZ, transition zone.

### 3.2 Distribution of TZ

Electroanatomic characteristics were presented in Table 2. The median TZ area was 9.4 (4.0–16.5) cm^2^ and the median TZ burden was 9.9 (4.2%–16.0%). The TZ burden was highest in the anterior wall (3.9 [1.2%–7.0%]), followed by septum (2.7 [1.3%–4.4%]). TZ was most frequently observed in the anterior wall (129, 81.6%) and septum (130, 82.3%), followed by posterior (83, 52.5%), roof (79, 50.0%), and inferior wall (79, 50.0%), while TZ occurrence in the lateral wall (18, 11.4%) was rare (Supporting Information S1: Figure 1).

**Table 2:** Electroanatomic and procedural characteristics.

	Total (*n* = 158)	TZ < 5% (*n* = 48)	TZ 5%−15% (*n* = 66)	TZ > 15% (*n* = 44)	*p* value
TZ, cm^2^	9.4 (4.0, 16.5)	2.4 (1.8, 3.9)	9.5 (7.1, 12.4)	21.3 (16.9, 25.4)	< 0.0001
TZ burden, %	9.9 (4.2, 16.0)	2.8 (1.8, 4.1)	10.1 (7.1, 12.2)	20.3 (16.8, 23.6)	< 0.0001
Anterior	3.9 (1.2, 7.0)	0.8 (0, 1.4)	4.4 (2.5, 6.3)	8.1 (6.3, 10.6)	
Posterior	0.7 (0, 1.8)	0 (0, 0)	0 (0.8, 1.7)	2.1 (0.9, 3.6)	
Roof	0.2 (0, 1.6)	0 (0, 0)	0.5 (0, 1.1)	1.7 (0.9, 2.3)	
Inferior	0.2 (0, 1.9)	0 (0, 0)	0 (0, 1.7)	2.4 (1.0, 3.9)	
Lateral	0 (0, 0)	0 (0, 0)	0 (0, 0)	0 (0, 1.4)	
Septal	2.7 (1.3, 4.4)	1.3 (0, 2.2)	2.4 (1.5, 3.4)	5.0 (4.0, 7.7)	
*Distribution of TZ, n (%)*					
Anterior	129 (81.6)	25 (52.1)	60 (90.9)	44 (100.0)	
Posterior	83 (52.5)	8 (16.7)	39 (59.1)	36 (81.8)	
Roof	79 (50.0)	10 (20.8)	34 (51.5)	35 (79.5)	
Inferior	79 (50.0)	11 (22.9)	32 (48.5)	36 (81.8)	
Lateral	18 (11.4)	0 (0)	5 (7.6)	13 (29.5)	
Septal	130 (82.3)	28 (58.3)	59 (89.4)	43 (97.7)	
Anterior LACV, m/s	1.25 ± 0.35	1.52 ± 0.36	1.21 ± 0.27	1.02 ± 0.25	< 0.0001
Mitral isthmus LACV, m/s	1.24 ± 0.20	1.23 ± 0.16	1.23 ± 0.23	1.26 ± 0.20	0.742
Inferior LACV, m/s	1.28 ± 0.19	1.29 ± 0.20	1.29 ± 0.18	1.25 ± 0.22	0.448
Total procedure time, min	136.2 ± 35.8	134.3 ± 32.0	135.8 ± 35.6	139.0 ± 40.3	0.814
Beginning to CPVI completion	106.0 ± 29.3	104.6 ± 26.3	106.0 ± 30.1	107.4 ± 31.7	0.901
CPVI completion to end	30.3 ± 18.0	29.7 ± 15.9	29.8 ± 17.6	31.6 ± 20.9	0.853
Total fluoroscopic time, min	9.0 ± 5.6	10.1 ± 5.3	8.5 ± 5.3	8.5 ± 6.1	0.261
Total RF delivery time, min	41.1 ± 19.2	39.4 ± 11.5	39.5 ± 20.0	45.4 ± 23.9	0.219
Electrical cardioversion	2 (1.3)	0 (0)	1 (1.5)	1 (2.3)	0.742

Abbreviations: CPVI, circumferential pulmonary vein isolation; LACV, left atrial conduction velocity; RF, radiofrequency; TZ, transition zone.

### 3.3 TZ and LACV

**Figure 4:**
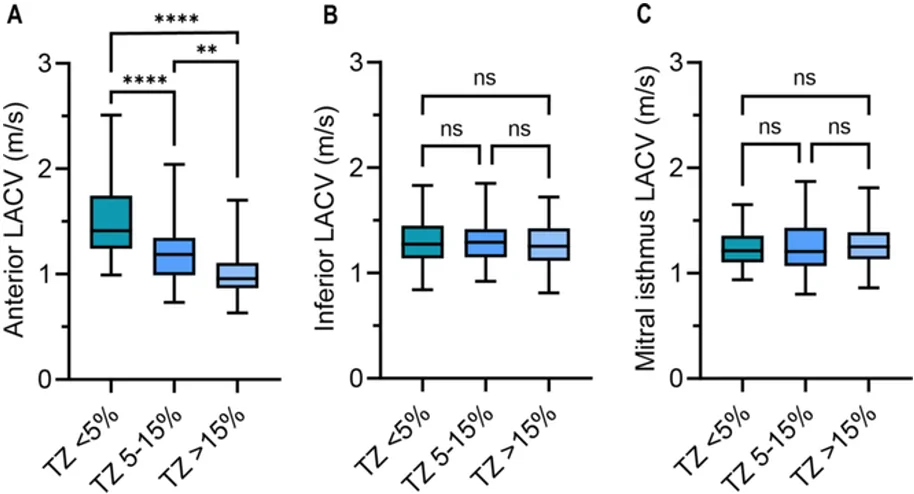
Comparison of (A) anterior, (B) mitral isthmus and (C) inferior LACV among 3 grades of TZ burden. LACV, left atrial conduction velocity; ns, no significance; TZ, transition zone; **p < 0.01; ****p < 0.0001.

The mean anterior LACV, mitral isthmus LACV, and inferior LACV were 1.25 ± 0.35 m/s, 1.24 ± 0.20 m/s, and 1.28 ± 0.19 m/s, respectively. Patients with higher TZ burden levels exhibited significantly decreased anterior LACV (1.52 ± 0.36 vs. 1.21 ± 0.27 vs. 1.02 ± 0.25 m/s, *p* < 0.0001), whereas no statistically significant differences were observed in mitral isthmus LACV (1.23 ± 0.16 vs. 1.23 ± 0.23 vs. 1.26 ± 0.20 m/s, *p* = 0.742) and inferior LACV (1.29 ± 0.20 vs. 1.29 ± 0.18 vs. 1.25 ± 0.22 m/s, *p* = 0.448) across the three groups (Table 2 and Figure 4).

Correlations between regional TZ burden and LACV are presented in Supporting Information S1: Table 1. Anterior TZ burden demonstrated the highest correlation with anterior LACV (*r* = −0.301, *p* < 0.0001), followed by septal TZ burden (*r* = −0.214, *p* = 0.008) and roof TZ burden (*r* = −0.161, *p* = 0.046). Lateral TZ burden showed the strongest correlation with mitral isthmus LACV (*r* = −0.143, *p* = 0.079), which was not statistically significant but very close. Only the inferior TZ burden exhibited significant correlation with inferior LACV (*r* = −0.226, *p* = 0.005).

### 3.4 TZ and Recurrence

**Figure 5:**
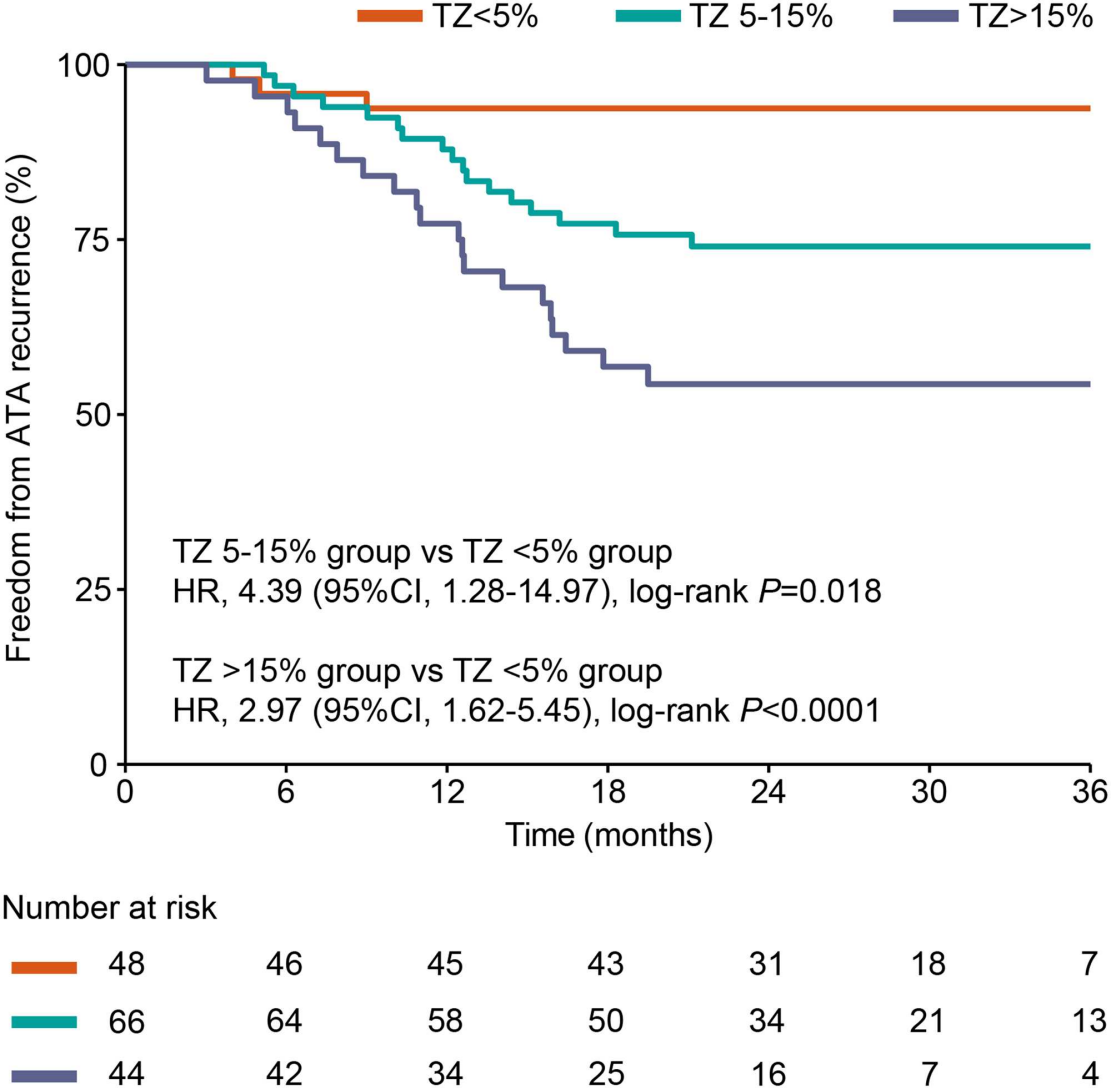
Kaplan−Meier curves for freedom from ATA after a single procedure among 3 grades of TZ burden. ATA, atrial tachyarrhythmia; CI, confidence interval; HR, hazard ratio; TZ, transition zone.

**Figure 6:**
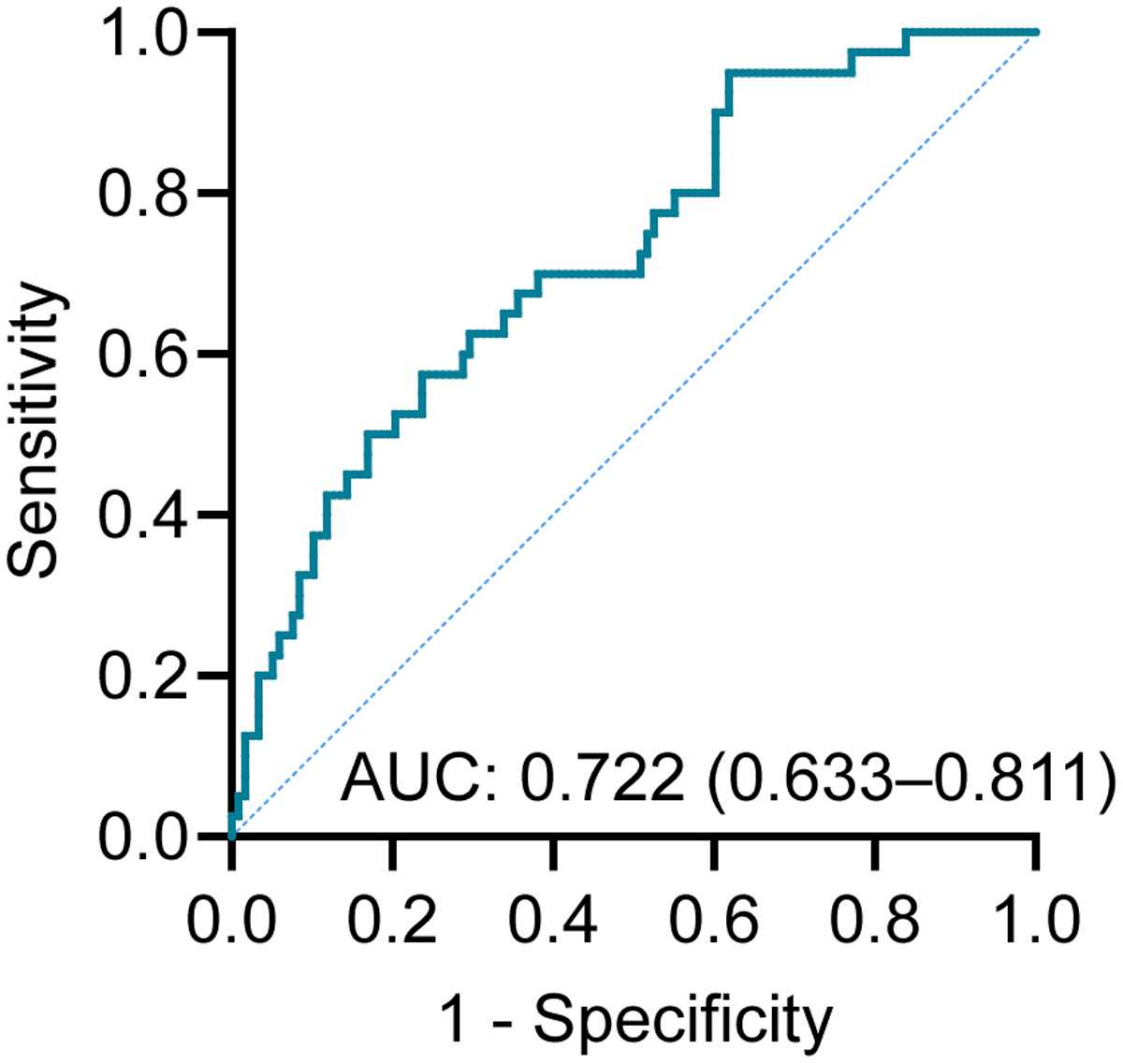
Receiver‐operating characteristic curve analysis for TZ burden to predict ATA recurrence after CPVI alone. The best cutoff value of TZ burden was 14.55% (sensitivity 58%, specificity 76%). ATA, atrial tachyarrhythmia; AUC, area under the curve; CI, confidence interval; CPVI, circumferential pulmonary vein isolation; TZ, transition zone.

Over a mean follow‐up of 24.9 ± 11.5 months, 40 (25.3%) patients experienced recurrence. Recurrent ATA occurred in 3 (6.3%) patients in the TZ < 5% group, 17 (25.8%) in the TZ 5%–15% group, and 20 (45.5%) in the TZ > 15% group. Kaplan−Meier analysis demonstrated a significant reduction in ATA recurrence for patients with a lower TZ burden group (Figure 5). Patients with recurrence had a significantly higher TZ area (15.1 [7.1–23.7] vs. 8.0 [3.6–13.9] cm^2^, *p* < 0.0001), TZ burden (15.4 [7.2–22.4] vs. 8.2 [3.5%–14.3%], *p* < 0.0001), and a slower anterior LACV (1.31 ± 0.35 vs. 1.06 ± 0.28 m/s, *p* < 0.0001) (Supporting Information S1: Table 2 and Supporting Information S1: Figure 2). ROC curve analysis demonstrated that TZ burden with a cutoff value of 14.55% best predicted ATA recurrence (AUC 0.722 [95% CI 0.633–0.811], sensitivity 58%, specificity 76%) (Figure 6). In the univariate COX regression analysis, only TZ burden was significantly associated with recurrence, and each 1% increase in TZ burden was associated with a 6.0% elevation in the hazard of AF recurrence over time (HR 1.060 [95% CI 1.032–1.088], *p* < 0.0001) (Table 3). After adjusting for demographics, cardiovascular risk factors, LAD and LVEF in a multivariable Cox model, this association remained significant (adjusted HR 1.065 [95% CI 1.034–1.098], *p* < 0.0001) (Table 4). Among 40 patients with recurrence, 25 patients underwent repeat ablation procedures. Pulmonary vein reconnection was identified in 20 cases (80%), and superior vena cava (SVC) triggers were detected in two cases (8%).

**Table 3:** Univariate and multivariable COX regression: clinical factors associated with arrhythmia recurrence.

Characteristics	Univariate COX regression analysis		Multivariate COX regression analysis	
	HR (95% CI)	*p* value	HR (95% CI)	*p* value
TZ burden, %	1.060 (1.032, 1.088)	< 0.0001	1.057 (1.028, 1.087)	< 0.0001
Female	0.825 (0.431, 1.580)	0.562		
Age, years	0.998 (0.921, 1.081)	0.953		
BMI, kg/m^2^	0.980 (0.888, 1.082)	0.693		
AF history, months	1.002 (0.999, 1.006)	0.221		
Hypertension	1.516 (0.741, 3.101)	0.255		
Diabetes	1.039 (0.436, 2.475)	0.931		
CAD	1.262 (0.601, 2.651)	0.539		
Stroke or TIA	0.758 (0.234, 2.457)	0.644		
LAD, mm	1.048 (0.985, 1.115)	0.136	1.014 (0.951, 1.081)	0.674
LVEF, %	1.022 (0.965, 1.082)	0.455		

Abbreviations: AF, atrial fibrillation; BMI, body mass index; CAD, coronary artery disease; CI, confidence interval; HR, hazard ratio; LAD, left atrial diameter; LVEF, left ventricular ejection fraction; TIA, transient ischemic attack; TZ, transition zone.

**Table 4:** COX regression analysis of TZ burden to predict atrial tachyarrhythmia recurrence adjusted for clinical features.

	Model 1		Model 2		Model 3	
	HR (95% CI)	*p* value	HR (95% CI)	*p* value	HR (95% CI)	*p* value
TZ burden, %	1.060 (1.032, 1.088)	< 0.0001	1.063 (1.035, 1.091)	< 0.0001	1.065 (1.034, 1.098)	< 0.0001

*Note:* Model 1: unadjusted model; Model 2: adjusted for age, gender and BMI; Model 3: adjusted for model 2 covariates plus baseline AF duration, hypertension, diabetes, CAD, stroke or TIA, congestive heart failure, COPD, OSAS, CHA2DS2‐VA score, LAD, LVEF.

Abbreviations: AF, atrial fibrillation; BMI, body mass index; CAD, coronary artery disease; CI, confidence interval; COPD, chronic obstructive pulmonary disease; HR, hazard ratio; LAD, left atrial diameter; LVEF, left ventricular ejection fraction; OSAS, obstructive sleep apnea syndrome; TIA, transient ischemic attack; TZ, transition zone.

## 4 Discussion

The major findings of this study are as follows: (1) TZ burden exhibited marked heterogeneity in the elderly patients with paroxysmal AF; (2) TZ was predominantly distributed in the LA anterior wall, while being nearly absent in the lateral wall in our maps; (3) LACV was inversely correlated with TZ burden along the corresponding conduction pathway, but not with TZ located outside the pathway; (4) TZ burden served as an independent predictor of recurrence following CPVI alone.

Atrial fibrosis reduces the quantity of activatable myocardial cells, leading to decreased amplitude of local bipolar electrograms [18]. TZ, defined as the transitional region between healthy and fibrotic myocardium, is highly associated with abnormal electrograms [10, 20]. In our previous study (mean age 55 years), the voltage range of the TZ was defined as 0.4–1.3 mV (EnSite) [10]. Based on six patients (mean age 44 years) without atrial arrhythmia, Yagishita et al. identified the TZ as regions with bipolar voltages between 0.5 and 1.1 mV (CARTO3) [20]. This discrepancy may be attributed to variations in the control population, mapping systems, electrode sizes, and inter‐electrode spacing. Yagishita et al. further reported a significant association between the presence of TZ and recurrence following CPVI in an AF cohort (*n* = 100, mean age 62 years) [20]. However, Bergont et al. found no such relationship in a larger population (*n* = 190) with a mean age of 58 years when defining the TZ as 0.5–1.0 mV (CARTO3) [21]. To ensure comparability with the STABLE‐SR‐III trial [16], we enrolled elderly paroxysmal AF patients without LVA as our study cohort. Given the age profile of our study population and the mapping system applied, we considered the threshold used by Bergont et al. to be more comparable. Therefore, the 0.5–1.0 mV range (CARTO3) was adopted as a pragmatic and literature‐consistent definition of TZ. Currently, the TZ burden thresholds for prognostic relevance have not been well established. To enable comparison, patients were categorized into subgroups of mild (< 5%), moderate (5%–15%), and severe (>15%) TZ burden, based on LVA burden stratification as described by Bergont et al. [21] In view of the arbitrariness of these thresholds, ROC curve analysis was already performed to determine the optimal cut‐off point for predicting recurrence. Additionally, TZ burden was analyzed as a continuous variable in the Cox regression model.

In this study, TZ is most frequently observed in the anterior wall and septum, consistent with the distribution pattern of LVA [10, 26]. Several mechanisms may account for the regional variation in TZ distribution: (1) external pressure from adjacent structures, such as the aorta (near the septum/anterior wall) [26]; (2) nonuniform atrial wall thickness, for instance, regions with thinner myocardium—such as the fossa ovalis—might display reduced voltage [17, 27]; (3) regional coronary perfusion differences, and (4) heterogeneous autonomic nerve innervation [26, 28].

Atrial fibrosis reduces gap junctions, lessens myocyte density, and increases interstitial collagen deposition, resulting in localized conduction slowing and enhanced anisotropy [18, 29, 30]. Previous studies have demonstrated significantly lower conduction velocity within LVAs compared with normal voltage regions [31]. However, despite their similar distribution patterns [28], the deceleration zones (DZs) and LVAs are not always spatially concordant [28, 32]. Our study extends this understanding by revealing an inverse relationship between LACV and TZ burden along the conduction pathway. Patients with higher global TZ burden exhibited significantly reduced anterior LACV, whereas conduction velocities in the lateral and inferior walls did not differ among groups. This observation aligns with the predominant distribution of TZ in the anterior wall. In patients with limited TZ burden (< 5%), the anterior LACV was significantly higher than that of the isthmus and inferior wall (*p* < 0.0001), possibly reflecting superior conduction via Bachmann's bundle [33, 34]. However, this conduction dominance was attenuated in patients with higher TZ burdens (5%–15% and >15%), suggesting that disproportionate anterior TZ accumulation compromises Bachmann's bundle conduction. The association between TZ and slow conduction partially explains the conduction delays detected in “healthy” regions lacking LVA. Notably, slow conduction and enhanced conduction heterogeneity have been demonstrated to facilitate reentry and rotor formation [32, 35], thereby promoting AF maintenance in patients with TZ.

Our findings demonstrate that TZ burden is heterogeneous in patients without LVA and independently predicts ATA recurrence following CPVI alone, underscoring TZ as a potential arrhythmogenic substrate. Therefore, additional ablation strategies targeting at TZ beyond CPVI may enhance single‐procedure success. In our study, TZ demonstrated preferential localization within the anterior wall and septum. However, the thin myocardium at the fossa ovalis may lead to low‐voltage recordings that do not consistently indicate atrial remodeling [27]. Concurrently, patients with elevated TZ burden exhibited significantly LACV reduction in the anterior wall. These findings implicate TZ in the anterior wall as the priority target for TZ modification. Furthermore, our cohort of elderly patients (65–80 years) exhibited higher TZ burden (9.9 [4.2–16.0] vs. 5.7 [1.5%–9.8%]) compared with a prior study in a younger population (mean age 58.8 ± 11.0 years) [21]. This observation is consistent with previous evidence showing a positive correlation between atrial fibrosis and age [36, 37]. In basis of AF‐induced atrial remodeling, elderly patients may have more potential atrial substrate (e.g., TZ) due to age‐related atrial degenerative changes and a higher burden of comorbidities compared to a younger cohort. Hence, elderly patients with PAF might particularly benefit from TZ modification. Overall, TZ‐targeted therapeutic strategies warrant further investigation in prospective studies.

## 5 Limitations

First, this is a single‐center and observational study focused on elderly patients with PAF, and the results may not be directly applicable to patients with LVA, persistent AF, or younger populations. Hence, external validation in broader cohorts is warranted to confirm the finding. Second, to facilitate comparison with prior studies, we adopted the same TZ voltage cutoff values without revalidating them in patients without ATA. Thresholds derived from younger populations may not be directly applicable to older patients and may partially overlap with age‐related physiological changes rather than purely pathological remodeling, which should be interpreted with appropriate caution. Given the limited sample sizes and exclusive reliance on electroanatomic mapping data in current studies, further investigations with larger cohorts and histological validation are required to establish more appropriate TZ cutoff values. Third, considering the potential impact of complex electrograms and anisotropic conduction within the TZ on measurements, we focused on global LACV rather than local conduction velocity within the TZ. Moreover, the frequent occurrence of double potentials in the septum may affect the assessment of LAT, which is why septal LACV was not measured in this study. Fourth, the thresholds to stratify TZ burden were applied as pragmatic categories to facilitate comparison, rather than implying biologically established TZ‐specific cut points. Nevertheless, significant prognostic differences across distinct TZ burden subgroups were observed, consistent with results of multivariate Cox regression analyses. Fifth, we used intermittent rhythm monitoring techniques rather than wearable devices or implantable loop recorders, which may have underestimated some asymptomatic recurrences.

## 6 Conclusions

TZ burden is associated with reduced LACV and independently predicts ATA recurrence following CPVI. Our findings suggest TZ as a potential arrhythmogenic substrate, providing insights for future prospective studies investigating TZ‐targeted ablation strategies.

## Conflicts of Interest

The authors declare no conflicts of interest.

## Supporting information


**Figure S1:** Incidence of TZ in each region of LA. LA, left atrium; TZ, transition zone.
**Figure S2:** Comparison of (A) TZ area, (B) TZ burden and (C) anterior LACV between patients without and with recurrence. LACV, left atrial conduction velocity; TZ, transition zone; ****p < 0.0001.
**Table S1:** Correlations between regional TZ burden and LACV.
**Table S2:** Comparison of baseline and electroanatomic characteristics between patients without and with recurrence.

## Data Availability

The data that support the findings of this study are available from the corresponding author upon reasonable request.
