# Supplementary material for: Association of Transition Zone Burden With Recurrence in Elderly Patients With Paroxysmal Atrial Fibrillation Without Low‐Voltage Areas
Source: Clin Cardiol. 2026 Aug 31;49(9):e70435. doi: 10.1002/clc.70435 (PMC13530383; doi:10.1002/clc.70435)
Supplement: Supplementary file 1 [file CLC-49-e70435-s001.docx]

Supplementary Material

**Table S1. Correlations between regional TZ burden and LACV.**

| **Regional TZ burden** | **Anterior LACV** | | **Mitral isthmus LACV** | | **Inferior LACV** | |
| --- | --- | --- | --- | --- | --- | --- |
|  | ***r*** | **P-value** | ***r*** | **P-value** | ***r*** | **P-value** |
| Anterior TZ, % | -0.301 | <0.0001 | -0.042 | 0.609 | 0.066 | 0.415 |
| Septal TZ, % | -0.214 | 0.008 | 0.100 | 0.221 | 0.039 | 0.636 |
| Roof TZ, % | -0.161 | 0.046 | -0.049 | 0.547 | -0.088 | 0.279 |
| Posterior TZ, % | -0.050 | 0.537 | 0.135 | 0.097 | 0.053 | 0.514 |
| Inferior TZ, % | 0.011 | 0.892 | 0.013 | 0.87 | -0.226 | 0.005 |
| Lateral TZ, % | 0.047 | 0.567 | -0.143 | 0.079 | 0.006 | 0.943 |

Values are Pearson correlation coefficients.

LACV, left atrial conduction velocity; TZ, transition zone.

**Table S2. Comparison of baseline and electroanatomic characteristics between patients without and with recurrence.**

| **Characteristics** | **Total (n=158)** | **Without recurrence (n=118)** | **With recurrence (n=40)** | **P-value** |
| --- | --- | --- | --- | --- |
| Female, n (%) | 61 (38.6) | 47 (39.8) | 14 (35.0) | 0.588 |
| Age, years | 69.7±3.8 | 69.7±3.9 | 69.7±3.6 | 0.995 |
| BMI, kg/m^2^ | 24.6±3.3 | 24.7±3.4 | 24.4±2.9 | 0.656 |
| AF history, months | 12.0 (5.0,39.0) | 12.0 (3.0,36.0) | 24.0 (6.0,84.0) | 0.121 |
| Comorbidities, n (%) |  |  |  |  |
| Hypertension | 107 (67.7) | 77 (65.3) | 30 (75.0) | 0.255 |
| Diabetes | 22 (13.9) | 16 (13.6) | 6 (15.0) | 0.82 |
| CAD | 29 (18.4) | 20 (16.9) | 9 (22.5) | 0.433 |
| Stroke or TIA | 15 (9.5) | 12 (10.2) | 3 (7.5) | 0.853 |
| Congestive heart failure | 1 (0.6) | 1 (0.8) | 0 (0) | 1 |
| COPD | 0 (0) | 0 (0) | 0 (0) |  |
| OSAS | 2 (1.3) | 1 (0.8) | 1 (2.5) | 0.443 |
| CHA_2_DS_2_-VASc score |  |  |  | 0.141 |
| 1, n (%) | 35 (22.2) | 29 (24.6) | 6 (15.0) |  |
| 2, n (%) | 63 (39.9) | 41 (34.7) | 22 (55.0) |  |
| 3, n (%) | 39 (24.7) | 32 (27.1) | 7 (17.5) |  |
| >3, n (%) | 21 (13.3) | 16 (13.6) | 5 (12.5) |  |
| LAD, mm | 37.7±5.2 | 37.3±5.2 | 38.9±5.0 | 0.092 |
| LVEF, % | 61.8±5.2 | 61.6±5.3 | 62.4±5.2 | 0.433 |
| TZ, cm^2^ | 9.4 (4.0,16.5) | 8.0 (3.6,13.9) | 15.1 (7.1,23.7) | <0.0001 |
| TZ burden, % | 9.9 (4.2,16.0) | 8.2 (3.5,14.3) | 15.4 (7.2,22.4) | <0.0001 |
| Anterior LACV, m/s | 1.25±0.35 | 1.31±0.35 | 1.06±0.28 | <0.0001 |
| Mitral isthmus LACV, m/s | 1.24±0.20 | 1.24±0.21 | 1.23±0.18 | 0.837 |
| Inferior LACV, m/s | 1.28±0.19 | 1.28±0.19 | 1.28±0.21 | 0.998 |
| Total procedure time, min | 136.2±35.8 | 136.9±37.3 | 134.5±31.2 | 0.714 |
| Beginning to CPVI completion | 106.0±29.3 | 107.4±30.6 | 101.9±24.7 | 0.305 |
| CPVI completion to end | 30.3±18.0 | 29.5±17.4 | 32.6±19.9 | 0.348 |
| Total fluoroscopic time, min | 9.0±5.6 | 9.1±5.4 | 8.6±6.0 | 0.678 |
| Total RF delivery time, min | 41.1±19.2 | 41.7±20.6 | 39.5±14.3 | 0.544 |

AF, atrial fibrillation; BMI, body mass index; CAD, coronary artery disease; COPD, chronic obstructive pulmonary disease; CPVI, circumferential pulmonary vein isolation; LACV, left atrial conduction velocity; LAD, left atrial diameter; LVEF, left ventricular ejection fraction; OSAS, obstructive sleep apnea syndrome; RF, radiofrequency; TIA, transient ischemic attack; TZ, transition zone.

**Figure S1. Incidence of TZ in each region of LA.** LA, left atrium; TZ, transition zone.

**Figure S2. Comparison of (A) TZ area, (B) TZ burden and (C) anterior LACV between patients without and with recurrence.** LACV, left atrial conduction velocity; TZ, transition zone; ****, P < 0.0001.
